# Years of potential life lost and productivity costs due to premature mortality from six priority diseases in Tanzania, 2006-2015

**DOI:** 10.1371/journal.pone.0234300

**Published:** 2020-06-09

**Authors:** Susan F. Rumisha, Janeth George, Veneranda M. Bwana, Leonard E. G. Mboera

**Affiliations:** 1 National Institute for Medical Research, Dar es Salaam, Tanzania; 2 SACIDS Foundation for One Health, Sokoine University of Agriculture, Morogoro, Tanzania; 3 National Institute for Medical Research, Amani Research Centre, Muheza, Tanzania; Ravensburg-Weingarten University of Applied Sciences, GERMANY

## Abstract

**Background:**

Mortality statistics are traditionally used to quantify the burden of disease and to determine the relative importance of the various causes of death. Some of the most frequently used indices to quantify the burden of disease are the years of potential life lost (YPLL) and years of potential productive life lost (YPPLL). These two measures reflect the mortality trends in younger age groups and they provide a more accurate picture of premature mortality. This study was carried out to determine YPLL, YPPLL and cost of productivity lost (CPL) due to premature mortality caused by selected causes of deaths in Tanzania.

**Methods and findings:**

Malaria, respiratory diseases, HIV/AIDS, tuberculosis, cancers and injuries were selected for this analysis. The number of deaths by sex and age groups were obtained from hospital death registers and ICD-10 reporting forms in 39 public hospitals in Tanzania, covering a period of 2006–2015. The life expectancy method and human capital approach were used to estimate the YPLL, YPPLL and CPL due to premature mortality. During 2006–2015, malaria, HIV/AIDS, tuberculosis, respiratory diseases, HIV+tuberculosis, cancer and injury were responsible for a total of 96,834 hospital deaths, of which 46.4% (n = 57,508) were among individuals in the productive age groups (15–64 years). The reported deaths contributed to 2,850,928 YPLL (female = 1,326,724; male = 1,524,205) with an average of 29 years per death. The average YPLL among females (32) was higher than among males (28). Malaria (YPLL = 38 per death) accounted for over one-third (35%) of the total YPLL. There was a significant increase in YPLL due to the selected underlying causes of death over the 10-year period. Deaths from the selected causes resulted into 1,207,499 YPPLL (average = 21 per death). Overall, HIV/AIDS contributed to the highest YPPLL (323,704), followed by malaria (243,490) and injuries (196,505). While there was a general decrease in YPPLL due to malaria, there was an increase of YPPLL due to HIV/AIDS, respiratory diseases, cancer and injuries during the 10-year period. The total CPL due to the six diseases was US$ 148,430,009 for 10 years. The overall CPL was higher among males than females by 29.1%. Over half (58%) of the losses were due to deaths among males. HIV/AIDS accounted for the largest (29.2%) CPL followed by malaria (17.8%) and respiratory diseases (14.6%). The CPL increased from US$11.4 million in 2006 to US$17.9 million in 2016.

**Conclusions:**

The YPLL, YPPLL and CPL due to premature death associated with the six diseases in Tanzania are substantially high. While malaria accounted for highest YPLL, HIV/AIDS accounted for highest YPPLL and CPL. The overall CPL was higher among males than among females. Setting resource allocation priorities to malaria, HIV/AIDS and respiratory diseases that are responsible for the majority of premature deaths could potentially reduce the costs of productivity loss in Tanzania.

## Introduction

Globally, mortality statistics, including crude and age-adjusted death rates, are traditionally used to quantify the burden of disease and determine the relative importance of various causes of death. However, these rates often do not describe the age changes in mortality [1], hence do not sufficiently quantify the burden of deaths that occur in young populations. Deaths among young individuals cause more loss of life years than the deaths among the older ones [2] which affects cost of productivity. Some of the most frequently used indices to quantify the burden of disease are years of potential life lost (YPLL) and years of potential productive life lost (YPPLL) [3]. These two measures are most often being used to reflect the mortality trends of younger age groups as they are likely to provide a more accurate picture of premature mortality [4], the impact of the disease and death, and the cost to the society [5]. Moreover, YPLL and YPPLL are preferred over the conventional measures because the latter do not quantify the extent of life years lost following early death [6].

By definition, premature deaths refer to deaths occurring to individuals before they reach the existing or currently known life-expectancy of a particular country [7]. The use of premature mortality measures such as the YPLL has the advantage in that it allows to selectively evaluate the leading causes of death in younger age groups [2, 8,9]. Such measures are likely to guide countries to define public health priorities and allocate resources to appropriate preventative interventions, focusing on the younger populations and deaths that could have been prevented [10]. Moreover, they provide a room for targeting the scarce resources available to high-risk areas and furthering investigation on the causes of premature death. YPLL is important to compare the relative impact of different diseases, to track differences in spatio-temporal trends, and to provide a framework for evaluating the cost-effectiveness of interventions [11]. On the other hand, YPPLL evaluates the loss incurred by the society measured in terms of the individual’s productive capacity in relation to the productive age population [12]. To quantify the value of labour productivity due to premature death, cost of productivity loss (CPL) is the most useful method to interpret the economic burden of disease and predicting costs to the society [13,14].

Health economists are most often concerned with measuring the impact of specific public health problems including diseases on individual, societal and national economy and using certain criteria or indicators to make judgments as regards whether it is worthwhile to invest in a certain area of public health [15]. In light of this view, any attempt to estimate the burden of specific disease events requires one to consider economic analyses as one of the key components in the measurement rather than being treated as just a supplement. Ignoring that, the horizon for which the analysis is eventually done cannot capture all the necessary parameters needed to come up with realistic and representative picture of the real world situation on the burden of disease in a broad and multifaceted epidemiological and economic context. As various analysts suggest, the evidence on the economic burden of diseases provide crucial basis for the decision makers to better allocate resources and target interventions to the population in need more efficiently and equitably [16–18]. The mortality indicators of the cost of the disease is often considered to account for the largest proportion of the total cost in determining the economic burden of a given disease [19,20].

In Africa, there are a few studies on economic burden of premature mortality on productivity loss attributed to most important causes of death [21,22]. To the best of our knowledge, there is no study that has quantified the cost of premature mortality in Tanzania at the national level using real and local data. In understanding the significance of the economic analyses and to inform priorities for disease control, this study was, therefore, carried out to estimate years of potential life lost, years of potential productive life lost and cost of productivity loss due to premature mortality from selected priority diseases in Tanzania.

## Methods

### General approach

Data used in this analysis were collected from 39 public hospitals in Tanzania including all levels, i.e. national, specialized, zonal, regional and district hospitals. All regions in Tanzania Mainland were included. The procedure for selecting study sites has been described in more details elsewhere [23–25]. Briefly, the regions were categorised into three strata based on their proportional contribution to the national population. Based on the 2012 census population statistics [26], the strata were low populated regions (Arusha, Geita, Iringa, Katavi, Kilimanjaro, Lindi, Manyara, Mara, Mtwara, Njombe, Pwani, Rukwa, Ruvuma, Shinyanga, Singida and Simiyu), medium populated regions (Kagera, Tabora, Morogoro, Kigoma, Dodoma and Tanga) and the high populated regions (Dar es Salaam, Mwanza and Mbeya). The distribution of the hospitals within the country and regions; epidemiological burden and spatial variations of malaria and HIV/AIDS endemicity; patterns of child mortality and human resource coverage were taken into consideration to ensure national representation. Based on the review, it was decided to include one hospital from each of the low populated regions; two hospitals from each of the medium populated region; and three hospitals from each of the high populated region. Finally, all the national, specialized, zonal referral and regional hospitals were purposely included in the study. Specialized hospitals included the Ocean Road Cancer Institute (ORCI), Muhimbili Orthopaedic Institute (MOI), Kibong’oto Infectious Disease Hospital and Mirembe National Psychiatric Hospital. In regions where the national or zonal referral hospital was included, the respective regional hospital was excluded. To obtain the needed number of hospitals for highly and medium populated regions, 10 district hospitals were included. These were randomly selected, for each region separately, excluding the district where the regional hospital was located. The selected hospitals make about a third of the public hospitals and 15% of all hospitals in the country.

The selection of the causes of death used in this analysis was based on the following criteria: HIV/AIDS, tuberculosis and malaria are three diseases that have received extensive international support in terms of resources (https://www.theglobalfund.org/en/); Respiratory diseases (other than tuberculosis) rank as the second largest killer diseases in Tanzania [23]; and, cancer and injury are the most important non-communicable diseases and emerging causes of death [27].

### Sources of data and data collection

Mortality data was extracted manually from death registers, inpatient registers and International Classification of Diseases (ICD-10) report forms. The research team and data collectors were trained on use of tools, including hospital registers and reporting forms, the types of data/variables required and ethical issues related to accessing medical records. A thorough search and compilation of all identified forms used to record mortality data was conducted. Data were extracted from each of the identified source by tracking each death to ensure completeness. This process was done until all death events that occurred and recorded in the hospital covering a period of 2006–2015 were collected. Data collected were the deceased’s age, sex, date and underlying cause of death. The details of the data collection processes have been described elsewhere [23].

### Estimation methods

The economic losses due to premature deaths for a 10-year period (2006–2015) were estimated using three different methods: years of potential life lost (YPLL), years of potential productive life lost (YPPLL) and cost of productivity loss (CPL). The analyses adopted two approaches, namely, life expectancy and human capital. The former was used to calculate YPLL while the latter was used to calculate CPL. Human capital approach places a monetary value on the loss due to ill health, disability or premature mortality using the present value of the expected future earnings. The analysis was enriched by information solicited from various sources. The productive age category in Tanzania is 15–64 years according to the National Bureau of Statistics [28]. The Tanzania population size for productive age group as per 2012 national population census [26] is 23,466,616 accounting for 52.2% of the total population. During the analysis, 60 years ceiling was adopted as the retirement age [29]. Cost of productivity lost was calculated using a 2016 Gross Domestic Product (GDP) per capita of US$ 968.79 [28]. In this analysis, we adopted a discount rate of 9% released by the Bank of Tanzania in 2017 [30]. The sensitivity analysis was done using 12% and 6% discount rates in order to adjust for any potential error. The life expectancy at birth was extracted from World Health Organization [31]. For males and females, life expectancies at birth are 60 and 64 years, respectively and the total is 61.8 years. Therefore, for comparison purposes, the total life expectancy (61.8 years) was adopted in order to ensure consistency.

The YPLL, YPPLL and CPL were estimated using the following formulas:

$$YPLL=\sum_{i=1}^{I}\left[\left({\text{number}\;\text{of}\;\text{cause-specific}\;\text{deaths}}_{i})\;\text{x}\;(\text{end}\;\text{point}\;\text{age}–{\text{midpoint}}_{i}\right)\right]$$

where *i* = 1,2,3, …, *I* are 5-year age groups for all population

$$\text{YPPLL}=\sum_{j=1}^{J}\left[({\text{number}\;\text{of}\;\text{cause-specific}\;\text{deaths}}_{j})\;\text{x}\;(\text{retirement}\;\text{age}–{\text{midpoint}}_{j})\right]$$

where *j* = 1,2,3, …, *J* are 5-year age groups for all productive population (15–64)

$$\text{CPL}=\sum_{\text{j}=\text{1}}^{\text{J}}\left({\text{YPPLL}}_{\text{j}})\;\text{x}\;\text{GDP}\;\text{per}\;\text{capita}\right)$$

CPL was thereafter adjusted by a discount rate to get the present value of the future costs. The figures were adjusted based on GDP per capita [12]. The Tanzania 2016 GDP per capita was used as reference. The future values were discounted at 9% in order to get present values because the arithmetic sum of lifetime earnings overstates the current year economic value of an individual [32]. The assumption was that the productive capacity of a person does not change with aging until the retirement age. For each death associated with the selected diseases, the total YPPLLs were estimated. The results are presented by year, sex and underlying cause of death.

To scale up estimates on these metrics at country level, we calculated the sampling weights based on the total hospital population by level and used that to calculate weighted deaths counts. We then re-calculated the YPLL, YPPLL and CLP using weighted death counts. In addition to this, we have calculated these metrics for all causes of death using weighted counts to determine the contribution of the seven causes of deaths used in our focus analysis in the total YPLL, YPPLL and CPL. The weighted deaths obtained were used to calculate “per unit” deaths for each hospital level included in the study. Using number of zonal, special, regional and district hospitals available in each region, we distributed the deaths accordingly then assessed the regional ranking of the metrics.

### Ethical considerations

This study received ethical approval from the Medical Research Coordinating Committee of the Tanzania National Institute for Medical Research (Ref. No. NIMR/HQ/R.8a/Vol. IX/2230). Permissions to access hospital registers and reporting documents were sought from the Ministry of Health, Community Development, Gender, Elderly and Children and the respective Regional Administrative Secretaries and Hospital Authorities. No informed consent was required in view of the retrospective nature of this study. All data were fully anonymized. No individual identifiable information like names of the deceased were extracted from the sources provided. However, all entries were given identification numbers.

## Results

### Mortality by age and cause

There were a total of 247,976 deaths during the 10-year period (2006–2015). However, the analysis was done based on 231,045 deaths with complete information of the deceased such as age and sex which are input variables. During the period under review (2006–2015) 41.9% (96,834) of the total deaths were caused by malaria, HIV/AIDS, respiratory diseases, tuberculosis (TB), HIV+TB co-morbidity, cancer and injury. Malaria was the main contributor of death accounting for 27.9% of the total, followed by respiratory diseases (22.1%), HIV/AIDS (17.6%), cancer (11.8%) and injury (10.9%). Mortality in children under 5 years accounted for about a quarter (24%) of the total deaths while a total of 57,508 deaths (59.4%) were reported among individuals in the productive age groups (15–64 years). Malaria and respiratory diseases accounted for 46.4% and 42.4% of the total deaths in children under 5 years, respectively (Table 1).

**Table 1:** Causes of deaths by the age category in hospitals of Tanzania, 2006–2015.

Age (years)	Cancer	HIV/AIDS	Injury	Malaria	Respiratory diseases	TB	HIV+TB	Total	% of the total
0–4	367	908	1,052	10,705	9,807	205	72	23,116	24%
5–9	278	221	434	1,810	526	94	41	3,404	4%
10–14	255	255	317	864	335	100	37	2,163	2%
15–19	266	306	519	795	321	152	50	2,409	2%
20–24	330	763	1,119	1,080	562	332	135	4,321	4%
25–29	445	1,815	1,456	1,379	996	576	274	6,941	7%
30–34	633	2,846	1,315	1,658	1,355	757	484	9,048	9%
35–39	810	2,912	982	1,519	1,354	796	538	8,911	9%
40–44	915	2,368	699	1,170	1,072	662	399	7,285	8%
45–49	976	1,672	560	989	933	606	288	6,024	6%
50–54	1,044	1,176	503	896	698	467	193	4,977	5%
55–59	934	747	342	652	503	362	118	3,658	4%
60–64	1,090	508	350	822	655	434	75	3,934	4%
65–69	829	243	213	546	455	278	40	2,604	3%
70–74	889	165	273	763	601	286	29	3,006	3%
75–79	577	71	164	446	384	180	16	1,838	2%
>80	806	84	282	891	850	270	12	3,195	3%
Total (%)	11,444 (11.8%)	17,060 (17.6%)	10,580 (10.9%)	26,985 (27.9%)	21,407 (22.1%)	6,557 (6.8%)	2,801 (2.9%)	96,834	

**Fig 1:**
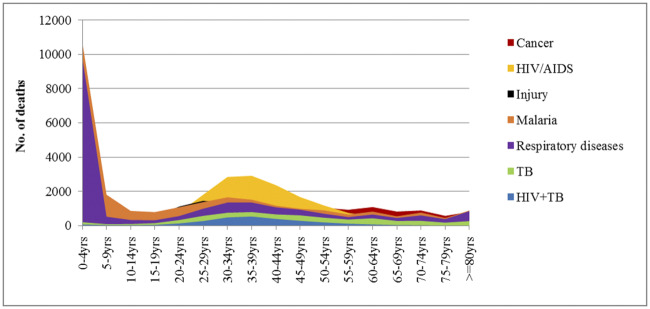
Distribution of cause-specific deaths by age category, 2006–2015.

A total of 44,939 (46.4%) deaths were reported among individuals in the productive age groups (15–64 years). HIV/AIDS was the main cause (75%) of deaths for the 20–54 years’ age group (Table 1, Fig 1). Injuries affected most the young adults (20–39 years) population. About half (51.8%) of the deaths due to tuberculosis affected the 25–49 years’ age groups. Cancer contributed to 27.7% of the total deaths in 60–64 years’ age group (Table 1).

### Mortality trend from 2006–2015

There was a general increase, with some variations, in mortality for all the selected underlying causes. However, the proportion of death due to malaria decreased significantly during the 10-year period (from 37% in 2006 to 21% in 2015); with a sharp gradient among males. Overall the contribution of HIV/AIDS to the total number of death ranged between 16–21% for the 10-year period. Deaths due to cancer and injuries rose at increasing rate (5% to 16%) and (7% to 13%), respectively during the period. Among the females, there was an increase in the number of cancer related deaths from 4% in 2006 to 17% in 2015. Among males, the major contributing causes of death were injuries (from 9% in 2006 to 18% in 2015) and cancer (from 5% in 2006 to 16% in 2015). The contribution of injuries to the total deaths increased significantly during the 10-year period. Cancers and HIV/AIDS associated deaths were more common among females than males.

### Years of potential life lost (YPLL) by specific cause

The reported 96,834 deaths accounted for a total of 2,850,928 years of potential life lost (YPLL) with an overall average of 29 years per death (female: YPLL = 1,326,724 [53%]; male: YPLL = 1,524,205 [47%]). On average, a death due to these causes had the highest YPLL among females (32 YPLL/death) than among males (28 YPLL/death). Malaria accounted for 35% followed respiratory diseases (28%), HIV/AIDS (15%), injury (11%), cancers (5%) and TB (4%). HIV/ AIDS accounted for higher YPLL among females (233,720) than among males (190,573) (Table 2). On the other hand, most losses due to injuries affected males (15%) than females (5%). Overall, malaria (38) and respiratory diseases (37) presented the highest YPLL per death followed by Injury (29) and HIV/AIDS (25). Tuberculosis (18) and cancer (12) had the lowest YPLL per death. Extrapolating the deaths at country level, the YPLL estimated from the seven causes of death was 17,903,344. A slight change on the YPLL contribution was observed particularly for malaria (45%) and cancers (1%) (Table 2).

**Table 2:** Estimates of years of potential life lost (YPLL) by cause of death by sex, 2006–2015 before and after extrapolation.

	Before extrapolation					After extrapolation				
Cause	Male	Per death	Female	Per death	Overall	Male	Per death	Female	Per death	Overall
HIV/AIDS	190,573	23	233,720	26	424,293	1,036,737	23	1,328,791	26	2,365,528
Malaria	531,997	37	478,196	39	1,010,193	4,217,950	38	3,765,880	40	7,983,830
Injury	228,611	28	72,413	30	301,024	1,238,522	29	339,566	31	1,578,088
Respiratory diseases	400,959	35	389,030	39	789,989	2,338,972	36	2,411,069	39	4,750,040
Cancer	65,807	11	72,190	14	137,996	114,373	9	136,701	13	251,074
Tuberculosis	71,606	17	48,903	22	120,509	372,938	16	298,746	22	671,684
HIV+TB	34,653	22	32,272	26	66,925	151,463	23	151,638	26	303,100
Total	**1,524,205**	**28**	**1,326,724**	**32**	**2,850,928**	**9,470,954**	**31**	**8,432,390**	**34**	**17,903,344**

**Fig 2:**
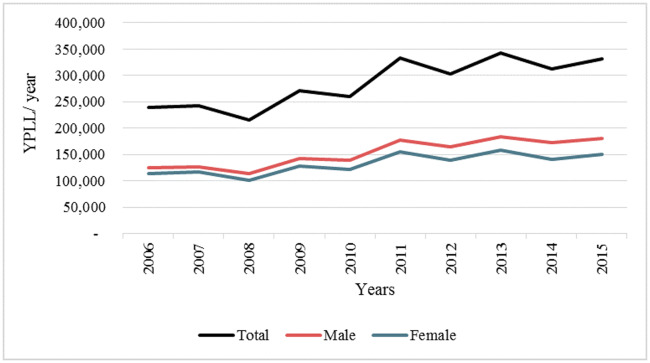
Estimates of years of potential life lost (YPLL) by sex, 2006–2015.

The overall trend indicates that there was significant increase in YPLL due to selected underlying causes of death over the period of 10 years. The male YPLL trend was slightly higher than that of the females (Fig 2), and the gap between the two sexes gradually increased with time. The YPLL in 2006 accounted for lowest (8%) of the total, while YPLL in 2015 accounted for highest (12%) indicating an increase of 4% during the 10-year period.

**Fig 3:**
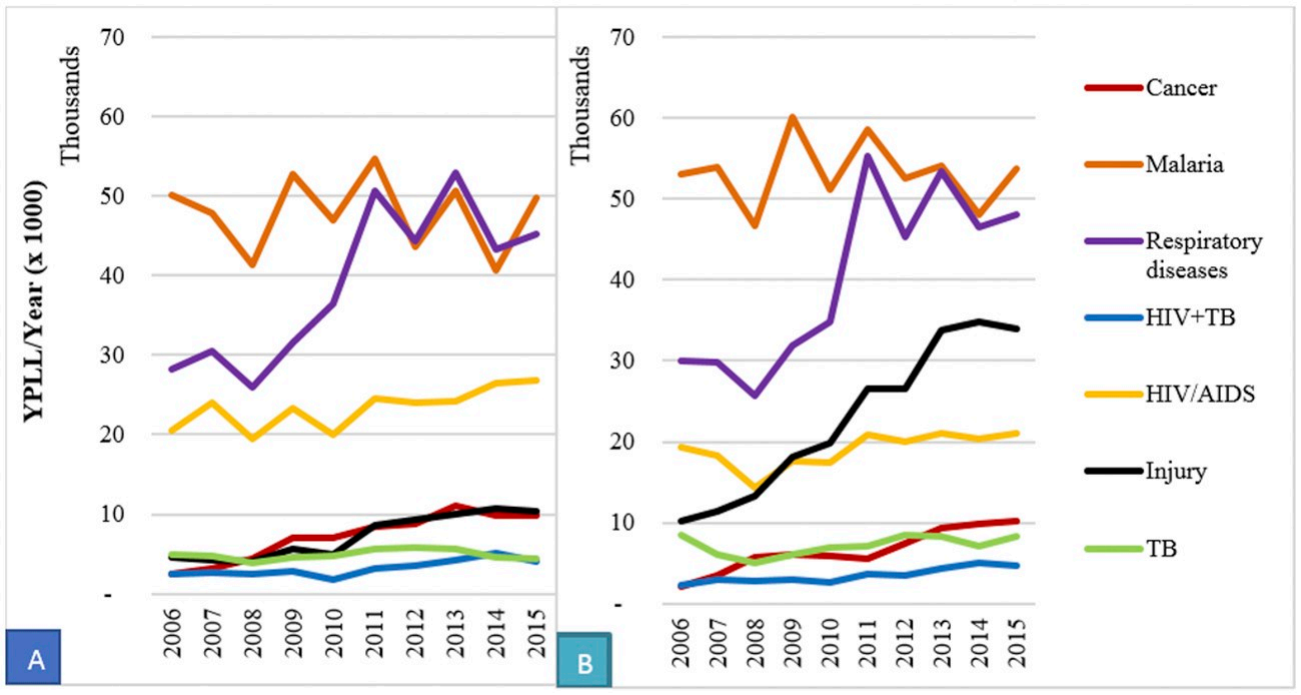
Cause-specific years of potential life lost (YPLL) trends for females (A) and males (B), 2006–2015.

When examining the death specific causes among males and females (Fig 3), YPLL due to malaria generally decreased from 2011 while respiratory diseases increased between 2010 and 2013. Among both males and females, YPLL due to respiratory diseases and malaria presented similar patterns. YPLL due to injuries among males increased from 2009 and stayed high throughout the period under review contrary to that of women which shows a gradual increase. For both sexes, YPLL due to cancer, HIV/AIDS, TB, and HIV+TB co-morbidity increased gradually with no much change observed with their values remaining in the lower bands during the 10-year period (Fig 3).

### Distribution of YPLL by geographical area

**Fig 4:**
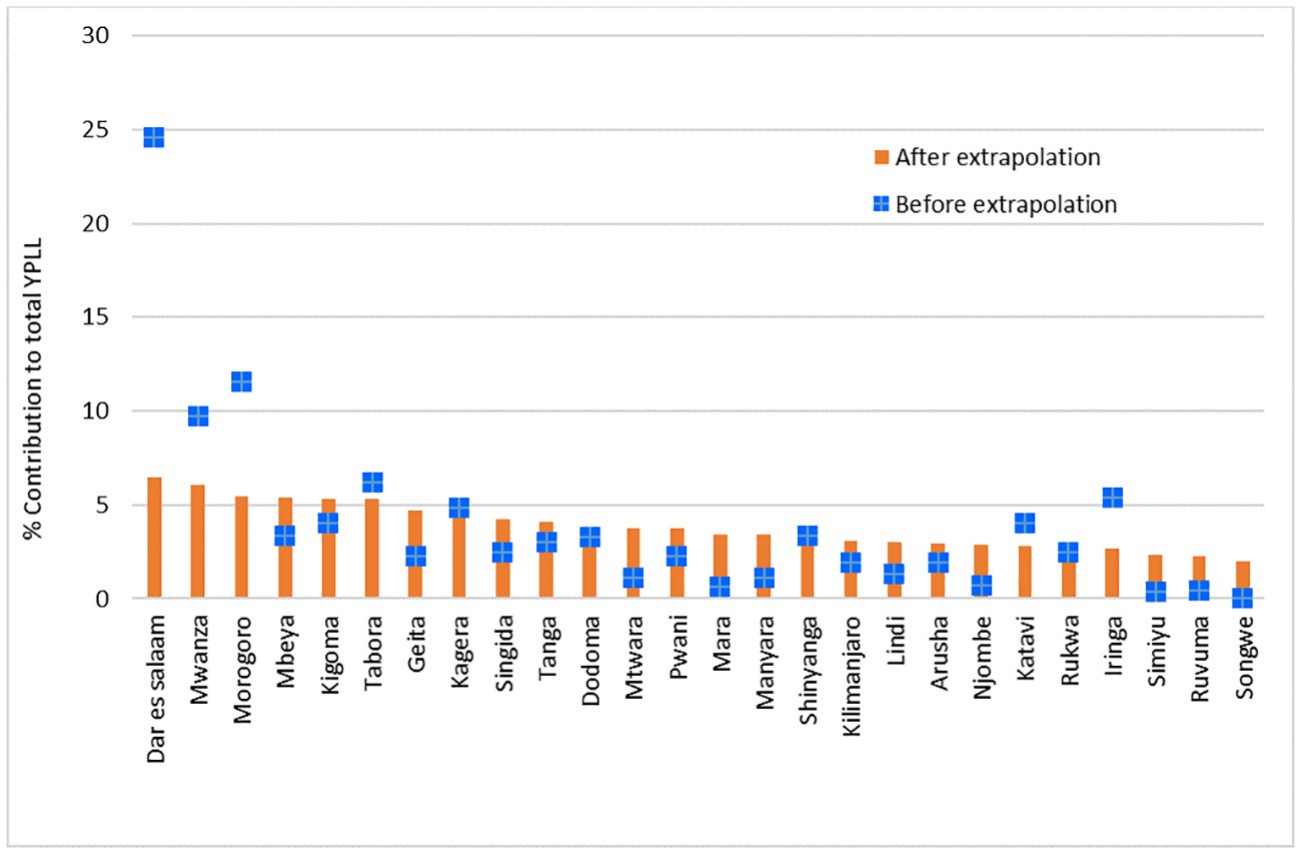
Regional distribution of the total years of potential life lost (YPLL), 2006–2015.

The distribution of YPLL per specific cause of death varied greatly across the regions. Dar es Salaam accounted for the highest YPLL (24.6%) followed by Morogoro (11.5%) and Mwanza (9.7%). High YPLL in these regions was attributed to the large number of deaths due to malaria, cancer, HIV/AIDS and injuries. Overall, Dar es Salaam registered highest YPLL for all causes of death, accounting for between 15 and 67% of the cause specific YPLL. Mwanza followed with relatively higher percentage in cancer (17%), HIV+TB co-morbidity (13%), HIV/AIDS (10%) and injuries (10%). Other regions which accounted for higher YPLL in specific causes included Morogoro for malaria and TB and Kilimanjaro for TB. Njombe, Ruvuma and Simiyu had the least YPLL because they had relatively fewer deaths compared to the rest of the regions (Fig 4). Scaling the deaths at country level revealed a different pattern in the contribution of the regions in the national YPLL, more homogeneity among regions was observed (Fig 4). Dar es Salaam, Mwanza and Morogoro still lead with high levels of YPLL, however, their contributions were comparable. Iringa which was previously ranked fifth indicates to have minimal contribution to the national level YPLL.

### Years of Potential Productive Life Lost (YPPLL)

A total of 57,508 individual belonging to the working age groups (15–64 years) died from the selected causes of which the majority (57%) were males. Deaths from the selected causes resulted into 1,207,499 YPPLL with an average of 21 YPPLL per death. Both sexes combined, HIV/AIDS contributed to highest YPPLL (27%, n = 323,704), followed by malaria (20%, n = 243,490) injuries (16%, n = 196,505) and respiratory diseases (14%, n = 174,317). Interestingly, on average a single death due to injury was equal to 25 YPPLL despite having relatively lower sum compared to malaria and HIV/AIDS, implying that more deaths belonged to the lower working age groups. Among the females, higher YPPLL were due to HIV/AIDS, malaria and respiratory diseases while among the males were injuries followed by malaria and HIV/AIDS (Table 3). After extrapolation, the YPPLL from these 7 causes was 6,942,278 with the average single death YPPLL increasing slightly from 21 to 23. HIV/AIDS and malaria carried equal burden of the national YPPLL (27%) while injuries and respiratory diseases contributed to 16% each (Table 3).

**Table 3:** Estimates of years of potential productivity life lost (YPPLL) by cause of death by sex, 2006–2015 before and after extrapolation.

Cause	Before extrapolation					After extrapolation				
	Male	Per death	Female	Per death	Overall	Male	Per death	Female	Per death	Overall
HIV/AIDS	139,665	20	184,039	23	323,704	782,739	20	1,074,369	23	1,857,108
Malaria	125,614	21	117,876	23	243,490	955,866	22	886,604	24	1,842,469
Injury	166,929	25	29,576	22	196,505	932,397	27	144,454	24	1,076,851
Respiratory diseases	83,878	19	90,439	23	174,317	512,186	19	603,786	23	1,115,972
Cancer	57,642	14	55,507	14	113,149	113,841	15	111,361	15	225,202
Tuberculosis	61,796	18	39,616	21	101,412	324,478	21	246,959	23	571,438
HIV+TB	29,039	20	25,883	23	54,922	129,312	21	123,926	23	253,238
**Total (%)**	**664,563**	**20**	**542,936**	22	1,207,499	3,750,819	21	3,191,457	23	6,942,277

**Fig 5:**
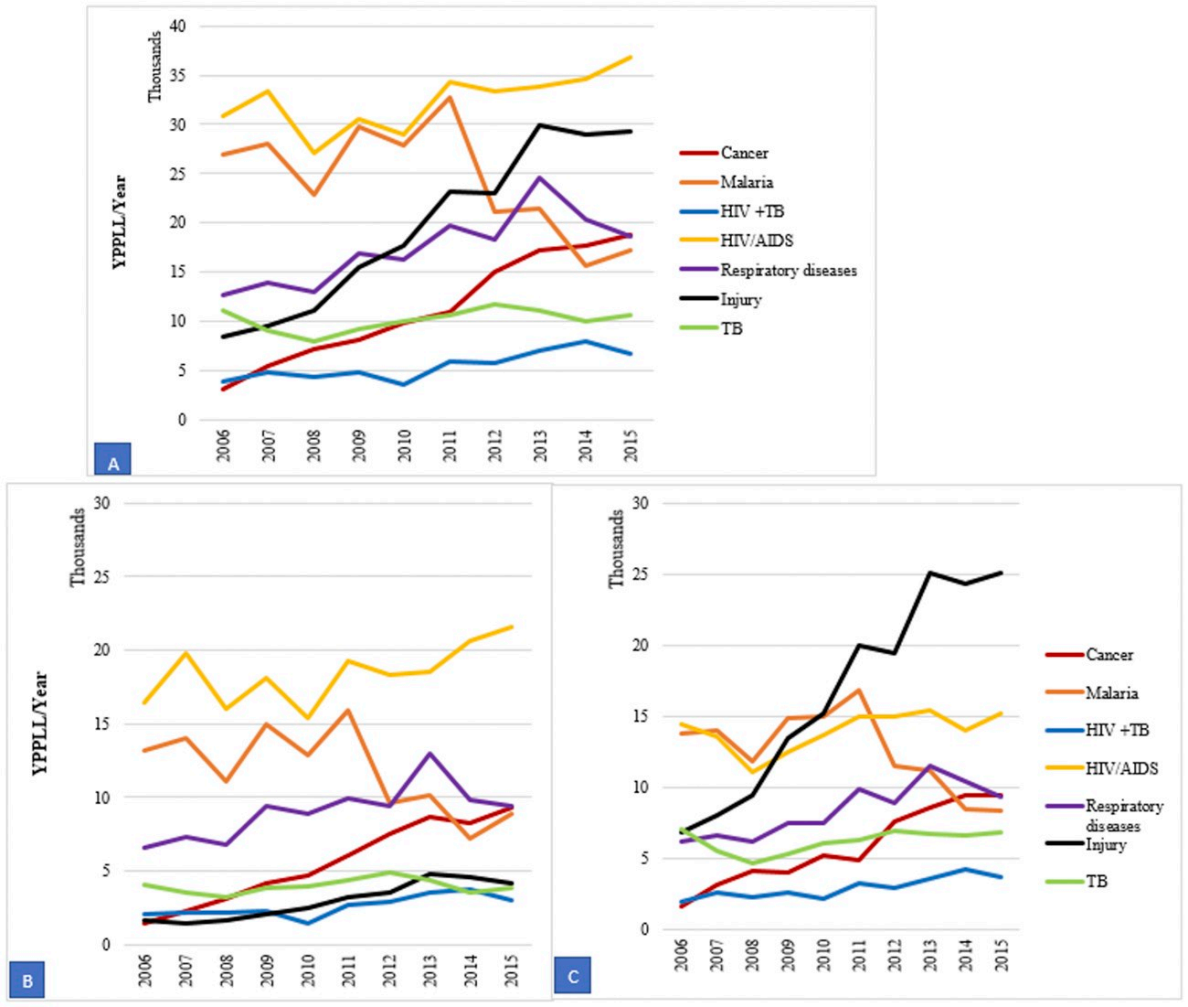
Trends in years of potential productivity life lost (YPPLL), 2006–2015. A is the overall trend of YPPLL, B is YPPLL for females and C is YPPLL for males.

Overall, males had higher YPPLL (664,563) than females (542,936). However, YPPLL per death due to HIV/AIDS was slightly higher among females (23) than males (20). Injuries related deaths contributed more to YPPLL among males than females (Table 3). There was a general decrease in YPPLL due to malaria (in both males and females) especially from 2012 to 2015 while YPPLL due to HIV/AIDS, respiratory diseases, cancer and injuries increased (Fig 5). The YPPLL for injuries increased markedly among males over the years while there was only a slight increase among females. The YPPLL due to respiratory diseases increased among males and females over the years. YPPLL for HIV+ TB co-morbidity has remained almost stable in both males and females for the entire 10-year period.

### Distribution of YPPLL by geographical area

**Fig 6:**
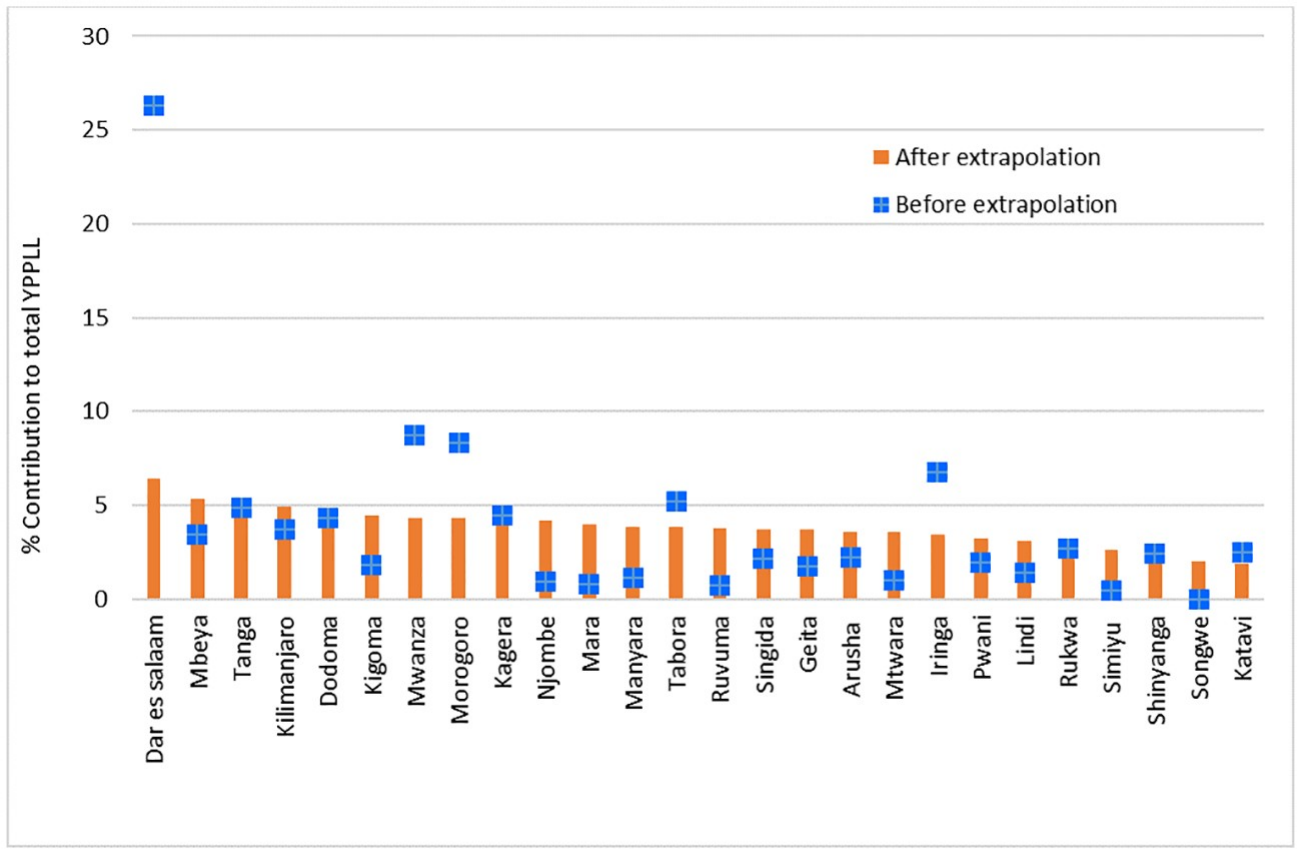
Percentage distribution of YPPLL by region before and after extrapolation.

From the total 1,207,499 YPPLL calculated from the deaths from the 7 causes, Dar es Salaam accounted for the largest proportion (26%) followed by Mwanza (8%) and Morogoro (8%). Simiyu (0.5%), Ruvuma (0.7%) and Mara (0.8%) had the lowest YPPLL (Fig 6). Dar es Salaam still accounted for higher losses for most causes of deaths (except for TB and HIV +TB), followed by Mwanza. YPPLL due to HIV +TB remained low in most of the regions. Scaling up the deaths at national level the ranking of regions in the distribution of YPPLL indicated a different pattern with much similarities between regions proportions. Dar es Salaam stills accounted for the highest proportion but not with an outlying high value. Regions such as Mbeya, Tanga and Kilimanjaro came into a play indicating slight higher contributions than Mwanza, Morogoro and Iringa which ranked higher in the sample. These patterns are partly explained by the variation of the age structure of deaths in these regions and number of hospitals of similar type presented in these regions.

**Fig 7:**
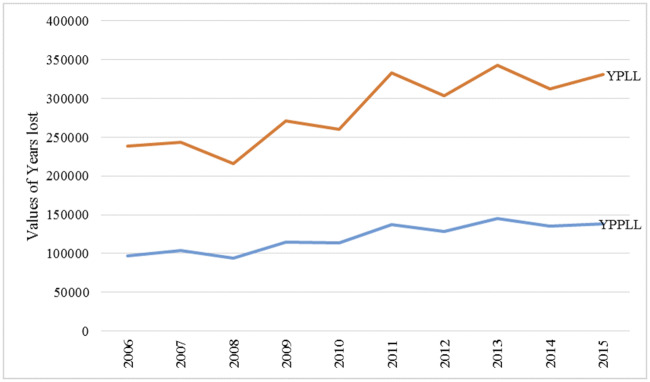
Comparison between YPLL and YPPLL trends.

When the values for YPLL and YPPLL were compared, YPLL was generally higher than YPPLL because it involved all the age groups and it has been increasing at higher rate (Fig 7). Furthermore, a sharp increase in YPPLL over the years was attributed to death due to injuries among males.

### Cost of Productivity Loss (CPL)

The total 10-year CPL caused by premature mortality due to the seven causes of death was US$148,430,009. Over half (58%) of the losses were due to deaths among males. HIV/AIDS accounted for the largest (29.2%) CPL followed by malaria (17.8%) and respiratory diseases (14.6%). The CPL was higher for HIV/AIDS and malaria among females and males, respectively. For deaths due to cancer, the CPL was higher for females than for males. CPL due to HIV+TB co-morbidity was higher among males than females. The CPL due to injuries was about six-fold higher among males than among females (Table 4). In the extrapolated results, HIV/AIDS accounted for the same percentage (29%), while that of malaria increased to 24% and cancer was reduced from 12% to 4%.

**Table 4:** Cost of productivity loss using GDP (in US$) by sex and underlying cause of death, 2006–2015 before and after extrapolation.

	Before extrapolation					After extrapolation				
Cause	Male (‘000’)	Per death	Female (‘000’)	Per death	Overall (‘000’)	Male (‘000’)	Per death	Female (‘000’)	Per death	Overall (‘000’)
HIV/AIDS	21,215	2,976	22,050	2,762	43,265	114,689	2,950	125,458	2,743	240,147
Malaria	14,561	2,444	11,838	2,367	26,398	109,173	2,458	87,437	2,394	196,610
Injury	15,877	2,419	2,826	2,205	18,703	83,558	2,389	12,618	2,127	96,176
Resp. diseases	11,569	2,599	10,101	2,526	21,670	69,550	2,588	68,313	2,560	137,863
Cancer	8,520	2,261	9,227	2,511	17,747	16,680	2,260	19,218	2,574	35,897
Tuberculosis	8,762	2,618	4,476	2,491	13,239	48,438	2,686	27,496	2,539	75,934
HIV+TB	4,286	2,991	3,121	2,785	7,408	18,482	2,979	15,353	2,842	33,835
**Total**	**84,790,717**	**2,597**	**63,639,292**	**2,560**	**148,430,009**	**460,569,799**	**2,606**	**355,892,213**	**2,568**	**816,462,012**

**Fig 8:**
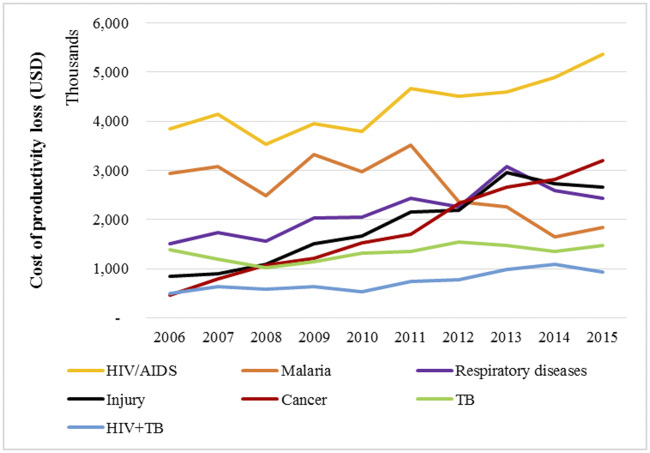
The trend in the cost of productivity loss for each of the selected diseases.

The CPL increased from US$ 11.4 million in 2006 to US$ 17.9 million in 2015. For specific causes, the CPL for HIV/AIDS and cancer increased markedly over the 10-year period. The CPL for malaria showed a marked decline from 2011 to 2015. CPLs for tuberculosis decreased slightly in 2008, but started rising slowly from 2009. CPL for respiratory diseases increased from 2006 to 2013, before decreasing in 2014 and 2015. The CPL for injuries increased sharply from 2006 to 2013 and dropped sligthly in 2014 and 2015 (Fig 8).

### Sensitivity analysis

Sensitivity analysis showed that the total CPL for the selected underlying causes of death were US$ 274,828,594 at 6% and US$86,062,638 at 12% discount rates. At 6% discount rate, HIV/AIDS accounted for the highest CPL (US$78,801,338) followed by malaria (US$50,562,221). Although the total CPL for HIV+TB co-morbidity was the lowest, it had the highest average cost per death for both males (US$ 5,296) and females (US$5247) (Table 5).

**Table 5:** Disease specific cost of productivity loss (in US$) using GDP per capita at 6% and 12 discount rates.

Discount rate	Cause	Male (‘000’)	CPL per death	Female (‘000’)	CPL per death	Total CPL (‘000’)
**6%**	HIV/AIDS	37,141	5,209	41,660	5,219	78,801
	Malaria	27,225	4,570	23,337	4,665	50,562
	Respiratory diseases	20,721	4,655	19,428	4,859	40,149
	Injury	31,867	4,856	5,622	4,385	37,489
	Cancer	14,725	3,907	15,504	4,220	30,229
	Tuberculosis	15,599	4,661	8,528	4,746	24,127
	HIV+ TB	7,589	5,296	5,882	5,247	13,471
	**Total**	**154,867**		**119,962**		**274,829**
**12%**	HIV/AIDS	12,875	1,806	12,474	1,563	25,350
	Malaria	8,408	1,411	6,487	1,297	14,895
	Respiratory diseases	6,875	1,545	5,619	1,405	12,494
	Cancer	5,251	1,393	5,819	1,584	11,070
	Injury	8,609	1,312	1,546	1,206	10,155
	Tuberculosis	5,235	1,564	2,522	1,403	7,757
	HIV+ TB	2,573	1,795	1,769	1,578	4,342
	**Total**	**49,826**		**36,237**		**86,063**

Using the GDP per capita, HIV/AIDS, malaria and respiratory diseases accounted for the higher CPL. The overall the CPL was higher among males than females by 24.9%. On specific causes of death, the CPL were higher among males than females for HIV+TB co-morbidity, injury, malaria and tuberculosis. Using the 12% discount rate, the CPL were generally higher for HIV/AIDS, malaria and respiratory diseases. However, the CPLs varied by sex and were relatively higher among males than females (Table 5).

## Discussion

In this study, malaria was the main contributor of premature death accounting for over a quarter of the total deaths, followed by respiratory diseases and HIV/AIDS. Overall, malaria and respiratory diseases accounted for the highest YPLL per death. Malaria accounted for slightly over 1 million YPLL contributing to a third of the total loss. HIV/ AIDS accounted for about 1.2 times higher loss among women than among men while most deaths due to injuries affected men than women. Recent global statistics indicate that HIV/AIDS and malaria are the leading cause of YPLL in Sub-Saharan Africa [22]. Higher YPLL ratios for malaria have also been reported in Angola, Republic of Congo, Equatorial Guinea, Gabon, Zambia and Tanzania [22]. The reported deaths associated with the selected diseases accounted for a total loss of over 2.8 million YPLL at an average of 29 years per death. The highest YPLL was observed among females than males. This can be explained by the lower age group at which the females died. Variations in YPLL among females and males have also been reported by other studies elsewhere [33,34]. The YPLL of 301,024 due to injuries in our study was lower than that reported in a study in Brazil [35], though the most affected age groups (20–29 years) were similar. The difference could be attributed to the differences in the end point age between the two studies.

There was a significant variation in the YPLL due to causes of death over the period under review. Examining the specific underlying causes of deaths, an increase or a decrease of specific cause YPLL was observed. The YPLL due to injuries, HIV/ AIDS, TB, and HIV+TB co-morbidity increased markedly over the 10-year period and varied widely across the regions. Overall, Dar es Salaam accounted for the highest YPLL followed by Morogoro and Mwanza. The higher YPLL for the three regions is explained by large number of deaths due to the selected causes of death. Njombe, Ruvuma and Simiyu regions had the least YPLL because they seemed to have recorded relatively fewer deaths as compared to the rest of the regions. Health outcomes have been described to vary at both the country and local levels and to be resultant from multiple causes [36,37], including the socioeconomic conditions or prevalence of risk factors and strength of the health system [38]. The fact that Dar es Salaam contributed about a quarter of the deaths can be explained as being attributed to the selection of the diseases that were included in this study. Most of the cancer cases and injuries were recorded from specialized hospitals located in Dar es Salaam [23].

As the data from our study reflect, males accounted for a significantly higher number of potential productive life loss than females. Unlike in the YPLL, HIV/AIDS contributed to the highest YPPLL, followed by malaria and injuries. On average injuries were responsible for the highest YPPLL (25 per death) among the selected causes. This indicates that injuries in Tanzania cause a substantial impact in terms of economic loss implying that most deaths affect the youngest working age groups. It has been described that economic loss to the society is heavier if a person dies at age 20 years, after the family has made full investment into the individual’s education and other costs of living [33]. Among the females, higher YPPLL were due to HIV/AIDS, malaria and respiratory diseases while among the males were due to injuries followed by malaria and HIV/AIDS. Gender disparities in injury mortality have been reported in other studies in Tanzania and elsewhere and were associated with the fact that males are at greater risk due to their lifestyle and behavioural risks [39–41]. Premature deaths due to HIV/AIDS was responsible for the larger number of productive years lost in Tanzania accounting for over a quarter of YPPLL. Deaths due to HIV/AIDS accounted for more YPPLL among females than males. This is likely to be associated with the fact that women acquire HIV infection at a relatively younger age than men [42]. On the other hand, injuries and TB associated deaths contributed more to YPPLL among males than females. There were significant variations in YPPLL due to cancer, malaria, respiratory diseases and HIV+TB co-morbidity between males and females. Higher YPPLL among males than females have also been reported in a recent study in Iran [43].

The estimated 1,207,499 YPPLL during the 10-year period resulted into a total loss of US$ 148.4 million. Over half of the losses were due to deaths among males. Overall, HIV/AIDS caused the highest cost followed by malaria and respiratory diseases. In our current study, there was a general decrease in YPPLL due to malaria especially from 2012 to 2015; most likely to have been contributed by the decline in the number of deaths caused by the disease [23]. On the other hand, YPPLL due to HIV/AIDS, respiratory diseases, cancer and injuries were increasing; indicating the contribution of young age individuals been most affected in recent years. When a cause or disease with low survival rate results in death in a young age, it generally receives higher rank of YPLL per death and cost per death [14, 18]. The cause-specific variations in YPPLL between males and females were remarkable as observed from the data presented from this study. Comparing the YPLL and YPPLL, data showed that the former (YPLL) was higher than the later, and this is because it involved all the age groups and it has been increasing at higher rate. Malaria accounted for highest YPLL because it affects mostly the younger age individuals while HIV/AIDS accounted for highest YPPLL because it affects most the young adults. Furthermore, there was a sharp increase in YPPLL due to injuries among males over the years.

In this study, the cost of productivity loss (CPL) due to cancer was relatively higher among females than males. Similarly, in a recent study in Iran, the total CPL due to cancer-related premature mortality was higher among males than females [17]. In contrary, a study in Australia reported that the costs for premature deaths from cancer were about three times higher in males than females due to the higher number of premature deaths in men, combined with higher levels of workforce participation and income [44,45]. In our study, the CPL estimates included all types of cancers; thus it is not easy to provide cost for specific cancer. Most previous studies elsewhere have produced estimates of productivity costs for all cancers or a single or small number of cancer sites [14,18]. The fact that during the 10-year period, CPL for malaria decreased markedly while that of HIV/AIDS increased, with negligible changes in the CPL for tuberculosis, indicate variations in the impact of the resources invested in the control of the three diseases. This is in contrary to the findings of a recent analysis which indicate that Global Funds disbursement for AIDS, Tuberculosis and Malaria was followed by accellerated reduction in all cause adult mortality due to HIV/AIDS [46]. There was a close relationship between malaria funding and decline in malaria specific under-five mortality [46]. In this study, though YPPLLs for HIV/AIDS and respiratory diseases were higher among females than males, their CPLs were relatively lower.

The CPL is sensitive to change in discount rate. By lowering the discount rate to 6% the CPL increased by 85% while 12% discount rate reduced the CPL by 42%. Total Tanzania’s GDP in 2016 was US$ 47.43 billion, thus in the current values, the cost amounts to 0.3% of the total GDP. This means that the higher the premature mortality especially of the productive age group, the higher the economic burden of the disease. It is worthwhile mentioning that this cost is only based on hospital mortality report, therefore, the figure would have definitely been higher if we included deaths that occurred outside health care facilities and morbidity costs before death.

This study has some limitations. Only public hospital inpatient data were included in this analysis, leaving out deaths that occur outside the health care facility and those from private hospitals. Moreover, misclassification of cause of deaths and under-reporting could lead to underestimation of mortality rates [24]. The fact that this analysis focused on productivity losses due to premature mortality, other productivity losses including early retirement and absenteeism from work were not considered.

In conclusion, the study showed that malaria, HIV/AIDS, respiratory diseases, cancers and injury impose a substantial burden in terms of premature deaths and productivity losses in Tanzania. The findings, also provide feedback on the impacts of interventions on major diseases such HIV/AIDS, malaria tuberculosis. Though it is still hard to measure economic impact at macro-economic level, the loss in manpower which could otherwise contribute to the national GDP if premature death could be prevented can be used as a proxy measure to the impact on the economic growth. Setting resources allocation priorities to malaria, HIV/AIDS, respiratory diseases and injuries that occur mostly among younger working-age individuals with high mortality rates could potentially decrease the YPLL and productivity losses to a great extent. Appropriate policies and interventions that prevent premature mortality due to these diseases would improve both health and economic outcomes. It is important that decision makers identify population and disease subgroups where cost-effective health care investment can achieve the greatest economic gains to society. Overall, there is need to strengthen the health care delivery system to reduce the premature deaths, most of which are preventable and effective interventions are available.
